# Calcinosis in a roe deer fawn (*Capreolus capreolus*) in northern Germany

**DOI:** 10.1186/s12917-020-02615-w

**Published:** 2020-10-28

**Authors:** Matthias Gerhard Wagener, Annika Lehmbecker, Melanie Bühler, Mirja Wilkens, Teresa Punsmann, Martin Ganter

**Affiliations:** 1 Clinic for Swine, Small Ruminants, Forensic Medicine and Ambulatory Service, University of Veterinary Medicine Hannover, Bischofsholer Damm 15, 30173 Hannover, Germany; 2 Institute for Pathology, University of Veterinary Medicine Hannover, Hannover, Germany; 3 Institute for Physiology and Cell Biology, University of Veterinary Medicine Hannover, Hannover, Germany

**Keywords:** Hyperphosphatemia, Azotemia, Renal failure, Vitamin D, Wildlife

## Abstract

**Background:**

Calcinosis has been reported for a broad range of different animals. Causes for calcinosis include metabolic disorders due to kidney failure, intoxication with calcinogenic plants, or iatrogenic overdose of vitamin D. Especially young animals seem to be very susceptible to developing calcinosis. Currently, however, there is a lack of information on calcinosis in wildlife.

**Case presentation:**

The following case report describes a roe deer fawn admitted to a clinic due to general weakness and myiasis. Plasma levels for creatinine, urea and phosphate were highly elevated, whereas the total calcium level was decreased. Necropsy revealed calcinosis due to calcification in many organs. The reason for calcinosis in this particular case might be kidney failure. Plasma samples from other hunted roe deer fawns also showed high phosphate levels.

**Conclusions:**

Roe deer fawns might be susceptible to calcinosis due to high plasma phosphate, which could be a result of kidney failure or different feed. Further research into calcium and phosphate homeostasis in roe deer is necessary.

## Background

Calcinosis can be induced by different metabolic disorders of the calcium phosphate homeostasis like tumour-associated hypercalcaemias, primary and tertiary hyperparathyroidism or iatrogenic hypercalcaemias [1]. Calcinosis due to vitamin D intoxication has been reported for different companion, farm and zoo animals, respectively [2–7]. Especially young animals as well as children seem to be very susceptible to developing calcinosis when vitamin D is overdosed [4, 5, 8, 9]. In ruminating animals, calcinosis can also be promoted by calcinogenic plants like *trisetum flavescens* which is indigenous in Germany [10, 11]. Different calcinogenic plants from other parts of the world like *Nierembergia veitchii*, *Nierembergia rivularis, Cestrum diurnum, Solanum malacoxylon, Solanum torvum, Solanum esuriale* or *Solanum vebascifolium* have also been reported to induce calcinosis in ruminants so far [12, 13]. The diagnosis “calcinosis” is often only made postmortem on the basis of macroscopically or histologically visible calcifications in various tissues. This case report deals with a case of severe calcinosis in a roe deer fawn with calcifications in several organs and is to our knowledge, the first reported case of such findings in a roe deer fawn. In contrast to other reports on wild animals, where often only a post-mortem diagnosis is possible, this report combines data from the blood profile with necropsical data.

## Case presentation

In July 2017, a (3.35 kg) female fawn of unknown age was presented to the Clinic for Swine, Small Ruminants and Forensic Medicine of the University of Veterinary Medicine Hannover, Foundation, Germany. The animal was found abandoned near a road in a local forest in the region of Hannover. Clinical examination revealed a poor body condition and myiasis in the perianal region. Auscultation of the lungs and the heart revealed no abnormalities. EDTA and lithium heparin blood samples were collected from the jugular vein for haematology and clinical chemistry (Table 1). Due to myiasis, the perianal region was cleaned and shaved to remove the fly maggots. Although the fawn was able to stand without assistance and suckled milk replacer for lambs, it died within two hours after being admitted to the clinic.

**Table 1:** Haematology and clinical chemistry

	Patient	Reference 1	Reference 1	Reference 2	Reference 3	Reference 4	Reference 5
**Haematology**							
PCV (L/L)	0.38	0.43–0.59	0.52 ± 0.005			0.53 ± 0.036	
WBC (G/L)	7.3	2.8–8.1	4.91 ± 0.23				2.92 ± 1.9
Haemoglobin (g/L)	153	150–204	185.91 ± 1.9			193.2 ± 28.2	
MCHC (g/L)	403	332–374	352.4 ± 1.39				
Lymphocytes (%)	17						54.2 ± 13.2
PMN (%)	57.5						36.4 ± 14.1
Band Neutrophils (%)	25						2.0 ± 1.0
Eosinophils (%)	0						4.4 ± 3.1
Basophils (%)	0						0.5 ± 0.6
Monocytes (%)	0.5						1.4 ± 0.9
Normoblasts (%)	0						
Lymphocytes (G/L)	1.24	1–4.1	2.4 ± 0.12				
PMN (G/L)	4.2	0.7–5.6	2.38 ± 0.21				
Band Neutrophils (G/L)	1.82	0–0.1	0.006 ± 0.004				
Eosinophils (G/L)	0	0–0.2	0.04 ± 0.01				
Basophils (G/L)	0	0–0.3	0.05 ± 0.01				
Monocytes	0.04	0–0.3	0.08 ± 0.01				
**Clinical chemistry**							
Total protein (g/L)	73.9	55–90	67.78 ± 1.51				
Albumin (g/L)	35.9	33.7–53-3	44.83 ± 4.47				
Albumin/Globulin	0.94						
CK (U/L)	804	60–822	442 ± 1451	978 ± 636			
Creatinine (µmol/L)	607	58–141	99.91 ± 2.13	136.9 ± 52.9			
Urea (mmol/L)	117.6	1.7–13.5	6.24 ± 0.37	6.3 ± 2.7			
Glucose (mmol/L)	8.39	6.6–18.2	10.52 ± 0.33				
Total calcium (mmol/L)	1.31			2.5 ± 0.3	2.54 ± 0.4		
Phosphate (mmol/L)	13.5	0.8–3.9	2.38 ± 0.07	4.1 ± 1.5	3.58 ± 0.5		
Sodium (mmol/L)	135.0	142–180	158.91 ± 0.76	143.9 ± 10			
Potassium (mmol/L)	5.4	3.3–5.9	4.34 ± 0.11				

Haematology revealed slight anaemia and mild leucocytosis with banded neutrophils. Clinical chemistry revealed severe uraemia with azotaemia, hypocalcaemia and hyperphosphataemia. Reference 1: [16] (Sweden); Reference 2: [14] (Slovenia); Reference 3: [33] (Germany); Reference 4: [34] (Germany); Reference 5: [15] (Germany)

Haematology revealed anaemia and mild leukocytosis with band neutrophils (Table 1). Plasma showed severe uraemia with azotaemia, hypocalcaemia and severe hyperphosphataemia (Table 1).

For a further characterisation of the calcium and phosphate homeostasis, we additionally determined parathyroid hormone: 158.4 pg/mL (Bovine Intact PTH ELISA Kit®, Immutopics Inc., San Clemente, CA, USA); 25OH-Vitamin D: 40.82 ng/mL (25(OH)-Vitamin D direct day ELISA®, Immundiagnostik AG, Bensheim, Germany); CrossLaps® (which is a parameter for bone-mobilisation): 3.473 ng/mL (Serum CrossLaps® (CTX-I) ELISA, Immunodiagnostic Systems GmbH (IDS GmbH), Frankfurt am Main, Germany).

Necropsy was performed at the Department of Pathology of the University of Veterinary Medicine Hannover, Foundation.

Macroscopically, the fawn showed ulcerative dermatitis around the anus and the vulva with evidence of a few maggots within the vulva, extending into the vagina. Associated multifocal, subcutaneous haemorrhages were detected. The kidneys were bilaterally enlarged and swollen. Furthermore, multiple 1 to 2 mm large whitish focal changes were seen in the myocardium. Endocrine organs were unremarkable.

## Histology

**Fig. 1:**
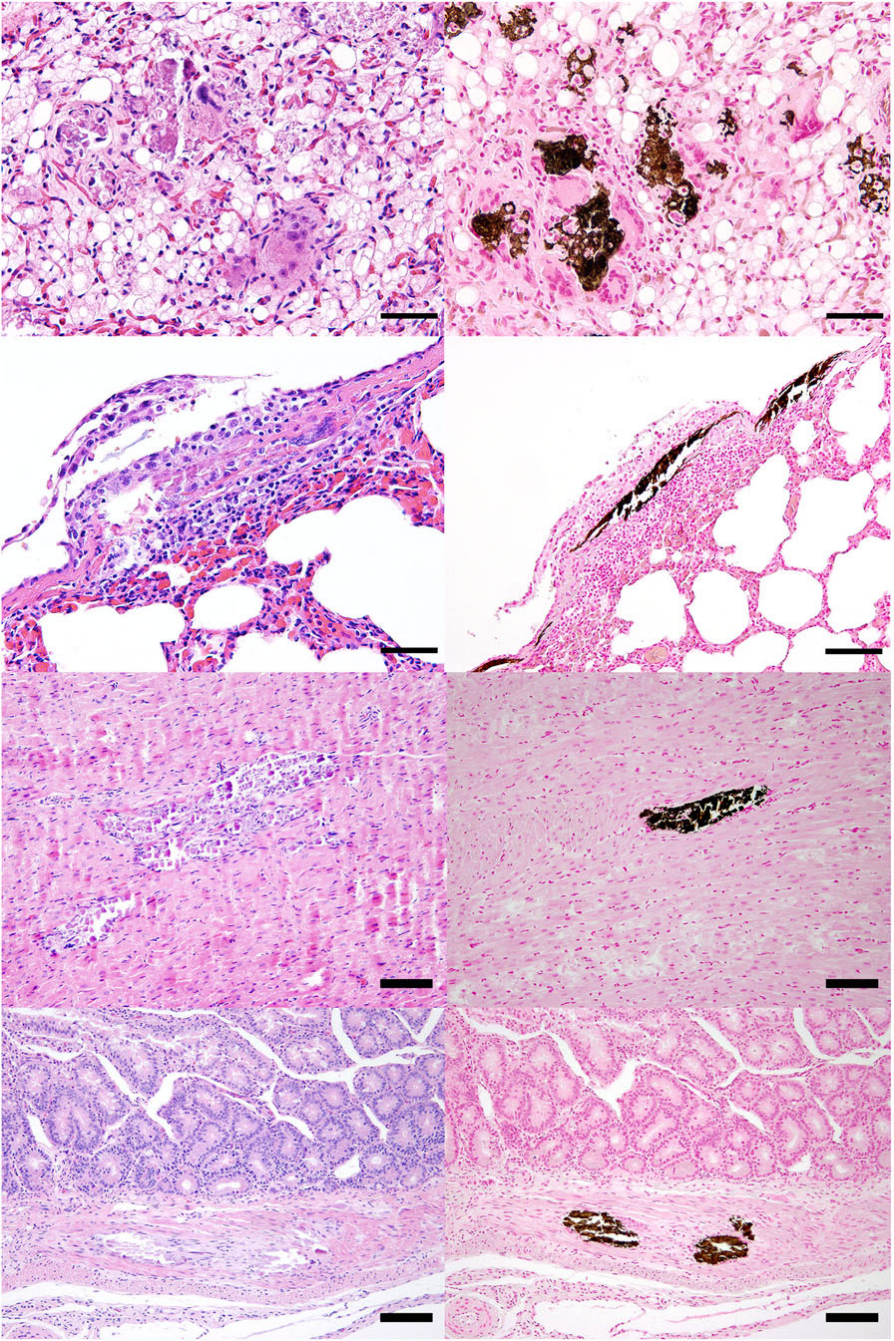
Histology of different organs. **a** (1st line, left): mediastinum (H&E): severe multifocal, granulomatous inflammation with multinucleated giant cells and intralesional deposition of an acellular, extracellular, basophilic material. **b** (1st line, right): mediastinum, von Kossa stain showing the extracellular material in brown-black, indicating mineralisation. **c** (2nd line, left): lung (H&E): subpleural granulomatous inflammation with intralesional mineralisation. **d** (2nd line, right): lung: von Kossa stain, showing intralesional mineralisation in brown-black. (**a**-**d**, bars = 50 µm). **e** (3rd line, left): myocardium (H&E): within the myocardium, granulomatous inflammation with intralesional mineralisation is evident. **f** (3rd line, right): von Kossa stain showing intramyocardial mineralisation. **g** (4th line, left): small intestine (H&E): intramural granulomatous inflammation with intralesional mineralisation. **h** (4th line, right): small intestine, von Kossa stain, intramural mineralisation shown in brown-black. (**e**-**h**, bars = 100 µm)

The maggots within the vulva were associated with a necrotising inflammation with high numbers of intralesional, coccoid bacteria. The regional, inguinal lymph node showed severe, suppurative inflammation and mild follicular hyperplasia. Bilaterally, the kidneys revealed moderate, multifocal, suppurative inflammation with dilated tubules and proteinaceous casts. The whitish areas within the myocardium were consistent with myocardial extracellular depositions of a basophilic, acellular material interpreted as mineralisation accompanied by mild, granulomatous inflammation (Fig. 1). Similar lesions were evident within skeletal muscles from various parts of the body (thigh, diaphragm, tongue, intercostal, fore leg) associated with mild to moderate, granulomatous inflammation. Furthermore, mineralisation was found to a great extent within the mediastinal adipose tissue, the thymus, within the splenic capsule, subpleurally within the lungs, and also within the intestinal wall and the adrenal glands (Fig. 1). Within the abomasum, a mild, multifocal, ulcerative inflammation was found. The small intestine revealed moderate multifocal neutrophilic inflammation (Fig. 1).

Representatively, a von Kossa stain was performed to confirm the intralesional deposition of phosphate within the mineralised areas (Fig. 1). To rule out a mycobacterial infection causing the granulomatous inflammation, a Ziehl–Neelsen stain was performed and no acid-fast bacteria were detectable.

Fluid from the anterior eye chamber was collected to evaluate the urea concentration (Urea test strips, Reference number 131299990350, DiaSys Diagnostic Systems GmbH, Holzheim, Germany). The urea content was severely elevated with > 300 mg/dL (reference value < 50 mg/dL).

## Discussion and conclusion

The cause of death of the fawn was renal failure with concurrent azotaemia and uraemia (Table 1) caused by suppurative nephritis originating from a haematogenous spread of bacteria. As portal of entry, the enteritis and also the necrotising dermatitis caused by the maggot infestation have to be taken into account. The upper reference range for white blood cells (WBC) differed depending on the source. Data from Zele et al. [14] and Drescher-Kaden and Hoppe [15] suggest leukocytosis in this animal, whereas the WBC would be classified as physiological when using the data from Küker et al. [16] (Table 1). Band neutrophils also indicate an ongoing inflammatory process, which might be associated with the inflammatory changes of the kidney and the severe myiasis. The uraemia of the animal caused by the kidney failure may have led to disturbances in skin integrity [17], which may have facilitated the infestation with fly maggots.

Additionally, the animal showed multifocal changes affecting several organs, deposition of von Kossa-positive materials, extracellular material interpreted as calcification. The cause for the dystrophic calcifications could be a disturbed calcium phophate homeostasis due to chronic renal failure, which has been reported for other species [18–20]. The elevated plasma levels of the fawn for creatinine, urea and phosphate and the decreased plasma level for calcium go in hand with blood values, that have been reported for young lambs suffering from nephropaties [21]. Under physiological conditions, the calculated product of calcium- and phosphate-plasma concentration in mammals ranges between 3.3 and 5.0 mmol/L [22]. The calculated product of calcium and phosphate in this animal was highly increased with 17.7 mmol/L. This led to systemic calcinosis by crystallisation of calcium phosphate in different organs, according to the pathological findings in this animal. The elevated plasma levels for phosphate, creatinine and urea and the mineralisation of different organs are also consistent with vitamin D intoxication in other mammalian species [2, 7]. However, evaluation of serum of four other roe deer (all female and younger than one year) hunted in January 2018 in northern Germany revealed no elevation of calcium, creatinine or urea levels. Levels for phosphate were in the upper reference range or slightly increased (Table 2). For roe deer, there are no comparable data available. However, for red deer, it was shown that younger animals had higher plasma levels for phosphate than adult animals (young red deer: 2.74–3.86 mmol/L; adult red deer: 0.82–2.16 mmol/L, respectively) [23]. In suckling goat kids, phosphate plasma levels on the 20^th^ and 50^th^ day of life were also found to be at a high level (3.33 ± 0.31 mmol/L on day 20 and 8.19 ± 0.37 mmol/L on day 50, respectively) [24]. Szabó et al. [25] detected significant differences in phosphate serum levels for farmed red deer yearlings depending on grass (phosphate: 2.1 ± 0.33 mmol/L) or papillonacceous (phosphate: 1.72 ± 0.24 mmol/L) pasture. Even though the investigated roe deer were young animals, the phosphate levels in the hunted roe deer with circa 5 mmol/L were much higher compared to those in the red deer (Table 2). These higher levels of phosphate in the hunted animal samples could be an indication of haemolysis in the blood, which was taken post mortem from the heart. Haemolysis in these cases was taken into account by measuring haemoglobin and using a correction factor. Another explanation might be an excessive intake of starch-rich food by the animals.

**Table 2:** Clinical chemistry of four healthy female roe deer (< one year) hunted in January 2018 in northern Germany

	**animal a**	**animal b**	**animal c**	**animal d**
Creatinine (µmol/L)	69	91	75	61
Urea (mmol/L)	9.18	5.68	6.26	13.36
Total calcium (mmol/L)	1.89	2.14	1.85	2.16
Phosphate (mmol/L)	5.89	5.23	5.29	4.89

The parameter CrossLaps® in this animal was much higher than in cows, goats or sheep [26–28], which might suggest high resorption of the bones in this fawn, which goes in hand with the severe hyperphosphataemia. The results for parathyroid hormone and 25OH-Vitamin D were in a similar range to those of cows [27]. Nonetheless, these data for parathyroid hormone, 25OH-Vitamin D and CrossLaps® have to be interpreted carefully. On the one hand, there are no reference ranges for roe deer available. On the other hand, these tests have not yet been established for roe deer and parameters were measured in haemolytic EDTA-plasma because there was no more lithium heparin blood available.

Intoxication due to *T. flavescens* in this animal cannot be excluded or confirmed since the plants found in the forestomach could not be differentiated. *T. flavescens*, in former years typically only growing in southern Germany near the Alps has recently spread to northern Germany and can also be found in more northern regions, where intoxications of horses have been reported recently [29]. *T. flavescens* was searched in the online database “Deutschlandflora-Portal”, which is offered by the “Network Phytodiversity of Germany”, in which the occurrence of plants in individual regions of Germany can be queried [11]. The database showed that *T. flavescens* is part of the local flora of the district where the roe deer fawn was found. Other calcinogenic plants that have been reported in the literature according to Mello et al. [12] could not be found in the database for this district. For sheep, Waser et al. [30] found that 1 kg dry substance *T. flavescens* had an effect comparable to 150,000 IU vitamin D. However, calcification of organs in the sheep used in this experiment could only be detected after 62 days of a daily intake of 24.4 g *T. flavescens* [30]. Simon et al. also described a dose-dependent effect of *T. flavescens* in sheep. After feeding sheep with diets containing varying amounts of *T. flavescens*, the sheep with the highest content of *T. flavescens* in the diet also suffered from the most severe mineralizations [31]. The age of the fawn was unknown, but in terms of date of discovery and body weight, it must have been between 4 and 8 weeks old. Therefore it seems rather unlikely that it has consumed large quantities of *T. flavescens* over such a long period.

Another explanation might be a vitamin D intoxication of the mother, which might have led to an intrauterine or postpartal alimentary intoxication of the fawn. Górniak et al. [32] showed in experiments with rats, that pups of dams, that were exposed to *S. malacoxylon* during pregnancy had increased levels for calcium and phosphate, which might be also possible in ruminants. In the present case, the health status of the mother remains unknown, because there were no data about the dam available, therefore this assumption also remains speculative. Iatrogenic vitamin D overdosage of the fawn seems unlikely because it was a wild animal with no signs of any clinical treatment before admission to the clinic. Dysregulation of calcium- and phosphate homeostasis due to hormonal dysbalances of calcitonin or parathyroid hormone seem unlikely, too, as no alterations of the thyreoidea or parathyroidea were found during necropsy.

In summary, the cause of calcinosis in this animal could not be clarified with absolute certainty. Most likely, inflammatory changes in the kidney seem to have led to serious disturbances in calcium-phosphate homeostasis with subsequent calcification of various organs. However, since similar changes are also observed after ingestion of *T. flavescens*, which grows in the fawn's area of origin, it is not possible to completely rule out golden oat poisoning as a possible cause for the calcifications. Not only the alterations in this fawn but also the high phosphate plasma levels in comparison with other species in the hunted animals (a-d) show that more research on calcium and phosphate homeostasis in roe deer is necessary.

## Data Availability

Not applicable.

## Abbreviations

H&E: Hematoxylin and eosin stain
EDTA: Ethylenediaminetetraacetic acid
WBC: White blood cells
PCV: Packed Cell Volume
PMN: Polymorphonuclear neutrophil
CK: Creatine kinase
